# 磺酸功能化共价有机骨架固相微萃取纤维的制备及其在小鼠脑部神经递质分析中的应用

**DOI:** 10.3724/SP.J.1123.2023.03006

**Published:** 2023-10-08

**Authors:** Cheng YANG, Yanmei SHI, Tiantian PANG, Xiaobing LIU, Zhiyu ZHANG, Kai HU, Shusheng ZHANG

**Affiliations:** 1.河南中医药大学中医药科学院, 河南 郑州 450046; 1. Academy of Chinese Medical Science, Henan University of Chinese Medicine, Zhengzhou 450046, China; 2.郑州大学现代分析与基因测序中心, 河南 郑州 450001; 2. Center of Advanced Analysis and Gene Sequencing, Zhengzhou University, Zhengzhou 450001, China

**Keywords:** 共价有机骨架, 固相微萃取, 超高效液相色谱-串联质谱, 神经递质, 海马区, covalent organic frameworks (COFs), solid phase microextraction (SPME), ultra performance liquid chromatography-tandem mass spectrometry (UPLC-MS/MS), neurotransmitters (NTs), hippocampus

## Abstract

神经递质(NTs)是细胞间交流的基本化学物质之一,研究表明,其含量的异常变化与多种神经性疾病相关。因此,建立精准的分析方法对神经递质的检测具有重要意义。本研究以三醛基间苯三酚和1,4-二氨基-2-硝基苯为共价有机骨架单体,经过衍生化后,在室温下制备了一种结晶性好、化学/热稳定性好、疏水性强、介孔结构均匀的磺酸功能化共价有机骨架材料(COF-SO_3_H)。然后,将其涂敷在不锈钢纤维上制备了具有强阳离子交换作用的新型涂层,利用扫描电镜、红外光谱、N_2_吸附-脱附等表征手段对COF-SO_3_H纤维的形貌、比表面积等性质进行表征,并比较了COF-SO_3_H纤维与HLB、C18、MCX、Amino、PXC 5种萃取纤维对神经递质的萃取效率,考察了相关参数对COF-SO_3_H萃取目标物的影响。结果表明,COF-SO_3_H具有良好的晶型,介孔结构分布均匀,与其他涂层材料相比,COF-SO_3_H纤维对神经递质表现出优异的萃取性能。最佳的萃取参数:脱附溶剂为甲酸-甲醇-水(0.5∶49.5∶50, v/v/v)、萃取时间和脱附时间均为15 min。在优化条件下,将COF-SO_3_H固相微萃取纤维与高效液相色谱-串联质谱法(UPLC-MS/MS)结合,建立了检测小鼠脑部神经递质的分析方法。7种目标神经递质在线性范围内具有良好的线性关系(相关系数*r*^2^均大于0.99),单胺类神经递质和氨基酸类神经递质的定量限(*S/N*≥5)分别为0.003~0.005 μg/mL和3~5 μg/mL,一日内连续制备4组同一质量浓度的样品进行分析,其4次检测的RSD低于20%, 7种目标神经递质在小鼠脑匀浆中3个水平下的精密度(0.80%~9.70%)和准确度(2.08%~17.72%)良好,绝对基质效应为82.22%~117.92%,表明复杂基质对目标分析物的准确测定影响较小。所建立的方法可以成功用于小鼠脑部目标神经递质的分离分析。

## Abstract (en)

Neurotransmitters (NTs) are essential for intercellular communication and primarily include monoamine, amino acid, and cholinergic NTs. These molecules play important roles in the body’s stress response, motor coordination, neuronal communication, and homeostatic functions. Previous studies have shown that abnormal changes in NT levels are associated with various neurological disorders. Therefore, the development of accurate analytical methods for NT detection will enhance the current understanding on complex neuropathophysiology by providing functional knowledge and techniques for early diagnosis, thereby facilitating the development of new therapeutic options for the related diseases. The solid phase microextraction (SPME) technique combines sample preparation, separation, and enrichment in a single step and is minimally invasive, low cost, solvent free, and high throughput. SPME has been successfully applied to the in vivo analysis of target analytes in animal, human, and plant tissues. The coating material plays a significant role in the development of in vivo SPME methods and must meet various analytical requirements, including a suitable geometry for the SPME device, high extraction capacity, excellent selectivity, and wide extraction coverage for the target analytes. Covalent organic frameworks (COFs) are porous crystalline polymers constructed from organic framework units through strong covalent bonds; these materials are characterized with a low density, large specific surface area, permanent porosity, excellent chemical/thermal stability, and easy functionalization.In this study, a sulfonic acid-functionalized COF material (COF-SO_3_H) with good crystallinity, excellent chemical/thermal stability, strong hydrophobicity, a uniform mesoporous structure, and narrow pore size distribution was prepared using 2,4,6-triformylphloroglucinol and 1,4-diamino-2-nitrobenzene as monomers. Then, the COF-SO_3_H was coated onto the surface of stainless-steel fibers and used for in vivo enrichment of NTs. The structural properties of COF-SO_3_H were characterized using various techniques, such as scanning electron microscopy (SEM), Fourier transform-infrared spectroscopy (FT-IR), and X-ray diffraction (XRD), all of which showed that COF-SO_3_H had a good crystalline structure and uniform mesopore distribution with a specific surface area of 46.17 m^2^/g. Compared with the SPME fibers of HLB, C18, MCX, amino, and PXC columns, the prepared COF-SO_3_H fibers showed better extraction efficiency for the target NTs. Next, the factors affecting SPME efficiency were optimized. The optimal desorption solvent was formic acid-methanol-water (0.5∶49.5∶50, v/v/v), and the optimal extraction and desorption times were 15 min. A method for the in vivo analysis of NTs in the brains of mice was established by combining the COF-SO_3_H fibers with ultra performance liquid chromatography-tandem mass spectrometry (UPLC-MS/MS) under optimal conditions. The NTs were separated on an Acquity UPLC BEH-C18 analytical column (100 mm×2.1 mm, 1.7 μm) with 0.1% formic acid aqueous solution (A) and acetonitrile (B) as the mobile phases. The flow rate was set to 0.2 mL/min, and the gradient elution procedure was as follows: 0-4 min, 5%B-6%B; 4-7 min, 6%B-5%B; 7-11 min, 5%B. Under optimal conditions, the method showed good linearity (*r*^2^>0.99). The limits of quantification (*S/N*≥5) were in the range of 0.003-0.005 μg/mL and 3-5 μg/mL for monoamine and amino acid NTs, respectively, with RSDs of less than 20%. The method showed good precision (0.80%-9.70%) and accuracy (2.08%-17.72%), with absolute matrix effects in the range of 82.22%-117.92%. These values reflect the good purification and enrichment abilities of the proposed fibers for the target analytes. Finally, the established SPME method was combined with UPLC-MS/MS and successfully applied to quantify target NTs in the brains of mice. The proposed strategy provides a practical method for the in vivo detection and quantitative analysis of NTs and expands the applications of functionalized COF materials for the analysis of various targets.

神经递质(neurotransmitters, NTs)是细胞间用来传递信息的基本化学物质之一,它们在机体对压力的反应、运动协调、精神运动控制以及神经元间的交流中起着核心作用^[1,2]^。这些化学物质根据结构差异分为单胺类神经递质(包括去甲肾上腺素、肾上腺素、多巴胺和5-羟色胺等)、胆碱类神经递质和氨基酸类神经递质(包括*γ*-氨基丁酸、谷氨酸和色氨酸等)。神经递质与其代谢产物参与人体的代谢、免疫和调节活动,其异常产生、释放和代谢与帕金森病、抑郁症、阿尔兹海默症、糖尿病以及肿瘤疾病等具有密切联系,神经递质可作为这些疾病的潜在生物标志物^[3⇓-5]^。对生物机体以及生物样品中的神经递质进行准确定量检测,可以为疾病的检测与治疗提供重要信息,反映药物疗效。因此,有必要开发有效的分析技术,对生物样品中的神经递质进行准确定量分析。目前,用于生物样本中神经递质检测的仪器分析方法主要有高效液相色谱法(HPLC)、电化学传感法、高效毛细管电泳法(HPCE)和液相色谱-质谱联用法(LC-MS)等^[6⇓-8]^。尽管目前用于检测神经递质的分析方法取得了一定进展,但神经递质在体内含量极低,基质干扰大,活体取样存在极大挑战。因此在分析时需要进行样品预处理从而达到富集目标物而消除基质效应的目的。

活体检测脑内神经递质含量变化对基础医学研究具有重要的意义。然而,由于待测组织的复杂性,在保证所需的分析性能的同时,还要保持待测组织的完整性,活体分析在实践中面临巨大的挑战。微透析技术是目前使用最广泛的活体取样技术之一,然而,微透析技术的透析液中存在的盐和缓冲液可能会对质谱检测产生影响。此外,微透析探针的植入可能会对神经组织造成损伤,引起神经递质含量的改变^[9]^。电化学方法虽然具有优异的时间分辨率和极高的灵敏度,但容易受到细胞外酸碱度变化的干扰。生物传感器不能同时用于多种神经递质的分析,并且由于蛋白质和其他代谢物的干扰,可能不适于复杂生物样品的分析^[10]^。固相微萃取(SPME)是一种集样品制备、分离和富集于一体的新技术,其克服了上述方法的缺点。体内SPME技术简化了分析过程中样本收集的步骤,减少了样品制备时间,更绿色环保^[11]^。SPME由于具有低侵入性、微创性、低成本、无溶剂、高通量等优点,已成功应用于动物、人类和植物组织的活体研究中^[12,13]^。例如,Reyes-Garcés等^[14]^利用SPME-HPLC-MS/MS技术研究了深度脑刺激后大鼠海马区的多种代谢物的变化。该研究使用了两种SPME纤维,C18/聚丙烯腈SPME纤维提供了强疏水相互作用,混合模式/聚丙烯腈SPME纤维提供了疏水相互作用和强阳离子交换作用。Aslam等^[15]^使用聚丙烯腈/反相酰胺SPME纤维研究了暴露于各种应激噪声条件下的大鼠两个脑区中阿那达胺和2-花生酰甘油的含量变化。研究表明,大鼠不同脑区对应激的反应不同,其不同脑区的阿那达胺和2-花生酰甘油的含量变化也不同,这可能是由于新环境引起脑部化学物质的变化,从而产生不同的应激反应。

SPME优异的性能为活体样品的分析提供了可行的新技术,然而,在体内SPME方法的开发中,所用的富集材料起着至关重要的作用。共价有机骨架(covalent organic frameworks, COFs)是一种由有机骨架单元通过强共价键构造而成的多孔结晶聚合物,具有低密度、高比表面积、永久孔隙率以及优异的化学/热稳定性和易于功能化的特点^[16,17]^。这些优点使COFs材料在气体储存和分离、催化、光电器件和化学传感器等领域都显示出巨大的应用潜力。此外,COFs在分析学科中也得到了越来越多的应用。在我们课题组的研究中,基于碳氮化物(MXene),通过原位生长的方式合成了COF@MXene复合材料,并将其用作SPME的涂层用于从蜂蜜样品中富集分离多环芳烃^[18]^。另外,我们课题组还通过共价键合的方法制备了基于三聚氰胺的磁性COFs材料(M-CTF-TPC),将该复合材料用作磁性固相萃取吸附剂,建立了减肥茶中蒽醌类化合物的分析方法,在优化条件下,所提出的方法得到了较低的检出限(0.010~0.077 μg/g)^[19]^。李慧等^[20]^报道了一种以亚胺连接的多孔共价有机骨架材料(IL-COF-1),该复合材料用做固相萃取吸附剂,结合HPLC-MS建立了快速检测蜂蜜样品中痕量雌激素的方法。Zhuang等^[21]^使用一种高效吸附剂TPB-DMTP-COF去除了水溶液中的磺胺嘧啶。除此之外,COFs材料也被用来富集分离生物样品中的神经递质。Wang等^[22]^制备了含氨基核壳结构的COFs(Fe_3_O_4_@TpBD(NH_2_)_2_),并利用2-甲酰基苯硼酸对其进行改性,合成了一种新型的磁性硼酸吸附剂(Fe_3_O_4_@COF@2-FPBA),使用该纳米复合材料在中性pH条件下富集了5种单胺类神经递质。在生物医学领域中,COFs材料也展现出了广泛的应用前景,包括药物递送、免疫治疗、肿瘤诊疗等。Zhou等^[23]^通过席夫碱缩合反应合成了COF@ICG@OVA纳米粒子,并将其作为光敏剂和载体。获得的COF@ICG@OVA纳米粒子经激光照射可以杀死癌细胞,同时释放抗原,激活机体的免疫应答,再进一步联合免疫检查点阻断疗法,阻断癌细胞逃逸。这些报道充分表明COFs材料具有良好的生物相容性,是富集神经递质的优质候选材料。

本研究选用具有强阳离子交换作用的磺酸功能化COFs涂层纤维(COF-SO_3_H)来富集活体小鼠脑部海马区的神经递质。COF-SO_3_H萃取纤维丰富的孔隙率、较大的比表面积增加了其与目标分析物的接触面积,缩短了扩散路径,增强了离子交换作用,实现了对目标神经递质的富集萃取。结合UPLC-MS/MS建立了活体脑组织中神经递质的富集分析方法,为神经递质活体检测和定量分析提供了一种切实可行的分析方法。

## 1 实验部分

### 1.1 仪器、试剂与材料

Thermo Ultimate 3000超高效液相色谱、TSQ Altis高分辨质谱仪、ESCALAB XI+X射线光电子谱、Nicolet iS20傅里叶红外光谱仪(美国Thermo Scientific公司); 3-18KS高速冷冻离心机(中国西格玛-奥德里奇有限公司); 2020HD88比表面及孔径分析仪(美国Micromeritics公司); JEM-2010F场发射扫描电子显微镜(日本电子株式会社(JEOL)); D2 PHASER X射线多晶衍射仪(XRD,北京布鲁克科技有限公司); HT7700透射电子显微镜(日本日立公司);脑立体定位仪(北京友诚嘉业生物科技有限公司)。

本实验使用的所有试剂均为分析级,三醛基间苯三酚、1,4-二氨基-2-硝基苯、四氢呋喃(THF)、甲酸(FA)、连二亚硫酸钠、1,3-丙磺酸内酯、去甲肾上腺素(NE)、肾上腺素(EP)、多巴胺(DA)、胆碱(Cho)、异丙醇(IPA)、乙醇(EtOH)、L-谷氨酸-^15^N (L-Glu-^15^N)、*β*-淀粉样多肽1-42 (*Aβ*1-42)均购自阿拉丁试剂有限公司(中国);乙酸(HAc)、*γ*-氨基丁酸(GABA)、谷氨酸(Glu)、5-羟色胺(5-HT)、氯化胆碱-三甲基-d_9_ (Cho-d_9_)、天麻素等均购自麦克林试剂有限公司(中国);聚丙烯腈(PAN)、甲苯、盐酸(HCl)、DA-1,1,2,2-d_4_、琼脂均购自西格玛-奥德里奇有限公司(中国);乙腈(ACN)、甲醇(MeOH)购自美国Fisher Scientific公司;*γ*-GABA-d_6_购自加拿大Toronto Research Chemicals公司。

药品制备:用生理盐水将*Aβ*1-42稀释至质量浓度为1 mg/mL,储存在37 ℃恒温培养箱中老化7 d,将老化后的*Aβ*1-42置于-20 ℃条件下备用。

### 1.2 色谱-质谱条件

Acquity UPLC BEH-C18色谱柱(100 mm×2.1 mm, 1.7 μm);流动相由0.1%甲酸水溶液(A)和乙腈(B)组成,流速为0.2 mL/min。梯度洗脱程序:0~4 min, 5%B~6%B; 4~7 min, 6%B~5%B; 7~11 min, 5%B。柱温为25 ℃,进样量为2 μL。

电喷雾电离源(ESI),利用选择反应监测扫描(SRM)模式进行正离子扫描,喷雾电压3.5 kV,毛细管温度350 ℃,加热温度325 ℃,鞘气25 arb,辅助通气10 arb,反吹气0 arb,氮气被用作碰撞气和C-trap的阻尼气。其他优化参数如表1所示。

**表 1:** 7种NTs及内标物的质谱参数

Compound	Precursor ion (m/z)	Fragment ion (m/z)	Collision energy/V	Radio frequency lens/V	IS
γ-Aminobutyric acid (GABA)	104.1	45.1	21.5	30	GABA-d_6_
	104.1	87.1	10.5	30	
Choline (Cho)	104.1	45.1	21.8	46	Cho-d_9_
	104.1	60.1	17.9	46	
Dopamine (DA)	137.1	91.1	17.9	47	DA-d_4_
	137.1	119.1	12.6	47	
Glutamate (Glu)	148.0	84.1	16.1	38	GABA-d_6_
	148.0	130.0	7.7	38	
Norepinephrine (NE)	152.1	107.1	18.1	61	L-Glu-^15^N
	152.1	135.0	12.3	61	
5-Hydroxytryptamine (5-HT)	160.1	115.1	23.2	69	L-Glu-^15^N
	160.1	132.1	17.3	69	
Epinephrine (EP)	166.1	107.1	19.6	72	DA-d_4_
	166.1	135.1	14.9	72	
γ-Aminobutyric acid-d_6_(GABA-d_6_)	110.2	93.1	10.7	30	
Dopamine-d_4_(DA-d_4_)	141.1	94.1	18.5	61	
L-Glutamine-^15^N (L-Glu-^15^N)	149.1	85.1	14.8	30	
Choline-d_9_(Cho-d_9_)	113.2	69.1	18.4	46	

### 1.3 COF-SO_3_H材料的制备

对文献[24]报道的COFs合成方法进行改进,合成COF-SO_3_H材料,其制备过程及萃取过程如图1所示。

**图 1:**
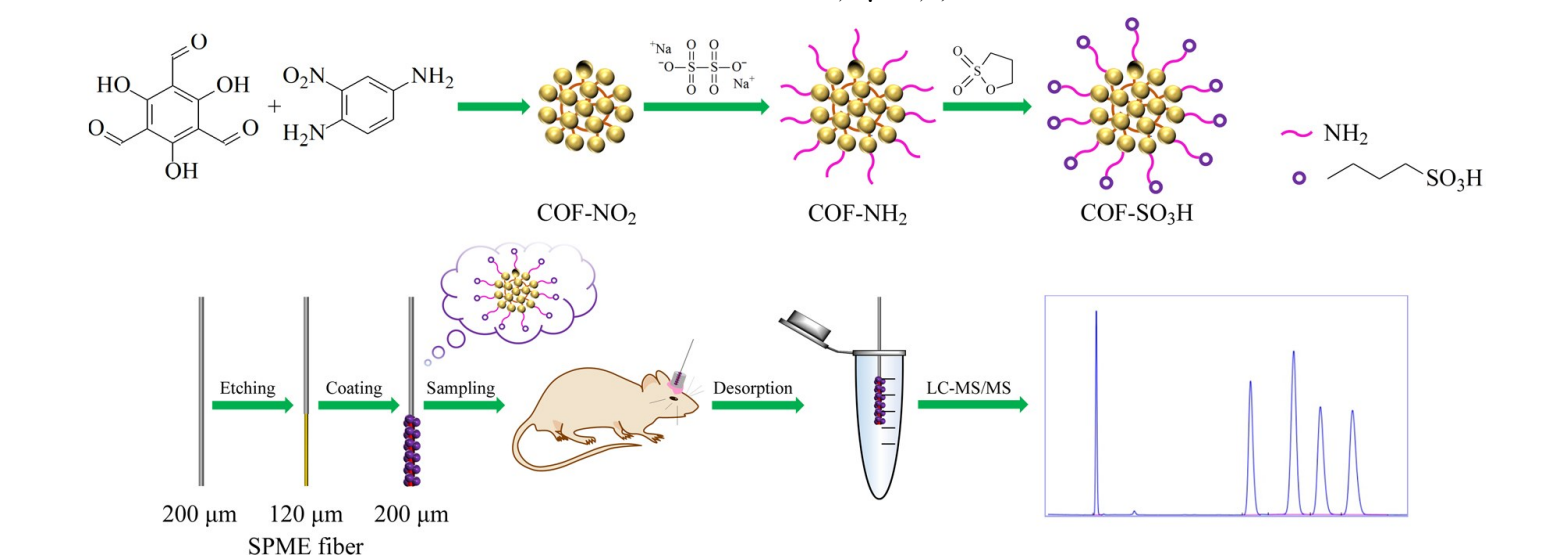
磺酸功能化COFs涂层纤维的制备流程及SPME过程图

COF-NO_2_的制备:将210 mg三醛基间苯三酚和230 mg 1,4-二氨基-2-硝基苯粉末分散于5 mL ACN中,超声混合溶解均匀后,再加入0.5 mL 12 mol/L HAc,振荡10 s混合均匀,将得到的混合溶液在室温下静置反应72 h。反应结束后,通过离心(1 min, 12000 r/min)收集得到红色沉淀,并分别用丙酮、THF和EtOH洗涤3次,60 ℃下真空干燥12 h后得到红色粉末状COF-NO_2_。

COF-NH_2_的制备:将600 mg COF-NO_2_置于100 mL圆底烧瓶中,加入3.6 g Na_2_S_2_O_4_、30 mL EtOH和6 mL H_2_O,将混合物在50 ℃下搅拌反应3 h。反应结束后,离心(1 min, 12000 r/min)收集沉淀,再分别用H_2_O和EtOH交替洗涤2次,除去未反应的Na_2_S_2_O_4_。最后,在60 ℃下真空干燥12 h后得到COF-NH_2_红色固体。

COF-SO_3_H的制备:将200 mg合成的COF-NH_2_加入到250 mL三颈烧瓶中,再加入100 mL无水甲苯和10 mL 1,3-丙磺酸内酯,装上冷凝装置,混合物在120 ℃下磁力搅拌12 h。反应结束后,用MeOH清洗材料,并在50 ℃下真空干燥12 h,得到COF-SO_3_H材料。

COF-SO_3_H萃取纤维的制备:取20 mg PAN和30 mg COF-SO_3_H材料加入0.3 mL *N*,*N*-二甲基甲酰胺(DMF),磁力搅拌4 h后,得到均匀的COF-SO_3_H悬浮液。将处理好的不锈钢丝蚀刻端(直径刻蚀120 μm)重复浸入装有COF-SO_3_H悬浮液的小瓶中,直至达到所需涂层厚度(涂层段的总直径(195±5) μm),涂层长度为2.5 mm。SPME纤维制备完成后,将其放入MeOH-ACN-IPA(40∶30∶30, v/v/v)的混合溶液中超声清洗20 min,除去在制造过程中产生的未固化的PAN胶和松散的聚合物颗粒。

### 1.4 小鼠脑替代基质制备

琼脂凝胶和生物体间质液的黏度非常相似。因此,使用琼脂凝胶模拟生物体间质液,用琼脂凝胶(琼脂与PBS缓冲液按质量与体积1∶50(g/mL)混合均匀)和鼠脑匀浆以体积∶质量2∶1(mL/g)的比例混合制备脑替代基质。

### 1.5 动物实验

KM小鼠(体重(35±5) g)购自济南朋悦实验动物繁育有限公司,饲养于温度(22±3) ℃、湿度55%±5%、每12 h明暗交替一次的清洁级环境中,自由摄食饮水,分笼喂养。本实验获得河南中医药大学实验动物伦理委员会的批准(No. DWLL202103189)。

在实验前喂养所有KM小鼠一周,以适应新的环境。挑选健康的小鼠随机分成对照组、模型组和天麻素给药组,每组6只。第8天所有小鼠通过手术进行造模并植入单侧留置导管。用异氟烷麻醉KM小鼠后,将其固定在脑立体定位仪上,保持颅骨水平,划开小鼠头部皮肤,剥离颅骨表面结缔组织。以前囟点(bregma)为0点,用颅骨钻0点向后2.5 mm、中线向右2 mm钻孔。微调微量注射器位置与钻孔位置相同,将连接微玻管的注射针缓慢下移2 mm。*Aβ*1-42溶液(模型组和给药组)或生理盐水溶液(对照组)于5 min内注射完毕,留针5 min以确保药物完全吸收分散。之后将定制的套管固定在孔中,在导管植入后,插入延伸至导管下方1 mm的探针,并拧紧防尘帽,以保持其清洁和通畅。在上述实验24 h后,给动物灌胃给药(正常组和模型组:生理盐水;给药组:天麻素,25 g/kg),一天一次,之后每周取样检测神经递质的含量变化。

### 1.6 活体SPME过程

将小鼠用异氟烷麻醉后,COF-SO_3_H萃取纤维通过留置导管插入小鼠头部,调整纤维深度,确保纤维萃取涂层完全进入小鼠脑部海马区后,静态萃取15 min后取出,将纤维用300 μL超纯水冲洗10 s,随后将其浸泡于100 μL的甲酸-甲醇-水(0.5∶49.5∶50,v/v/v)中超声解吸15 min,将解吸液进行UPLC-MS/MS分析。

## 2 结果与讨论

### 2.1 COF-SO_3_H材料和COF-SO_3_H萃取纤维表征

COF-SO_3_H SPME纤维的横截面SEM形貌如图2a所示,可以看出COF-SO_3_H SPME纤维中COF-SO_3_H分散在由PAN聚合形成的孔中,纤维涂层的厚度约为40 μm。图2b为纤维表面的SEM图,可以看到纤维表面有丰富的孔隙。此外,相应的元素映射分析显示,COF-SO_3_H材料中C、O、N、S元素分布均匀,C含量为60.06%, O含量为13.77%, N含量为24.94%, S含量为1.24%。

**图 2:**
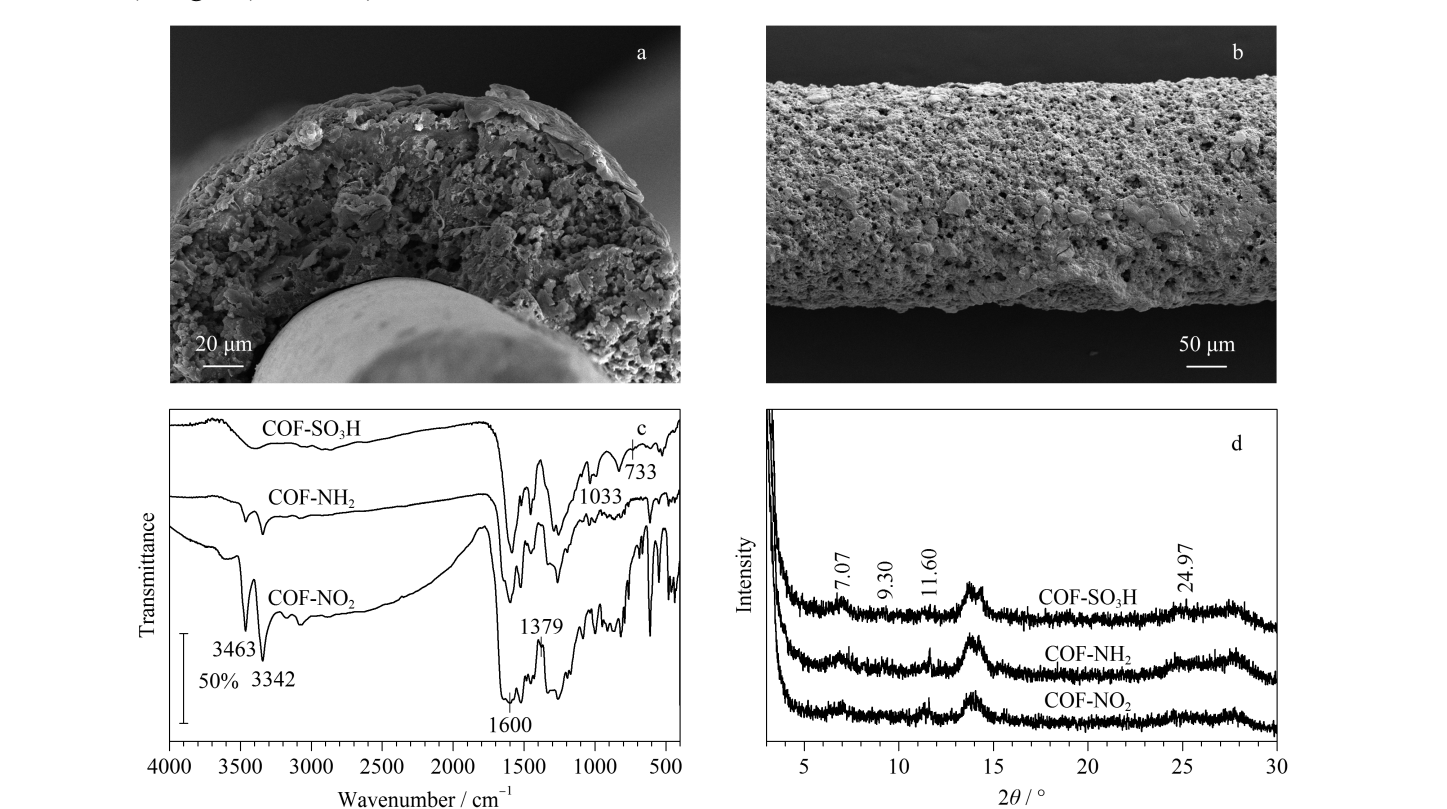
COF-SO_3_H纤维(a)横截面和(b)表面的SEM图及其材料的(c)FT-IR光谱和(d)XRD表征图

利用红外光谱对制备的COF-NO_2_、COF-NH_2_和COF-SO_3_H进行官能团结构表征。如图2c所示,COF-NO_2_的红外图谱中在1600 cm^-1^和1379 cm^-1^处出现了硝基吸收峰,在3342 cm^-1^和3463 cm^-1^处出现了氨基的特征吸收峰。COF-NO_2_被Na_2_S_2_O_4_还原成COF-NH_2_后,硝基的特征峰消失,伯胺的特征吸收峰减弱。在COF-SO_3_H的图谱中发现了1033 cm^-1^处的S=O伸缩振动峰和733 cm^-1^处的C-S伸缩振动峰,结合SEM和元素映射分析结果,表明COF-SO_3_H材料的成功合成。用XRD对制备的COF-SO_3_H的晶型进行研究。如图2d所示,3种COF材料在7.07°、9.30°、11.6°和24.97°处有弱峰,结果表明合成的材料为典型的COFs结构。

通过测量77 K下的N_2_吸附-解吸等温线来研究球形COFs的多孔结构,结果显示,吸附剂的氮吸附等温线与Ⅳ等温线一致,表明材料主要具有介孔结构。然后基于Brunauer-Emmett-Teller模型分析比表面积,COF-SO_3_H的比表面积为46.17 m^2^/g,在*p/p*_0_=0.9900时计算的孔体积为0.38 cm^3^/g,根据BJH(Barrett-Joyner-Halenda)方法计算孔的平均直径为33.2 nm。

### 2.2 萃取条件优化

#### 2.2.1 6种萃取纤维萃取效果的比较

首先,考察了所制备的COF-SO_3_H萃取纤维与HLB、C18、MCX、Amino、PXC 5种不同类型的萃取纤维对目标神经递质的萃取效果。根据萃取到的所有神经递质的相对含量对萃取纤维进行评估。由于每种萃取纤维的性能不同,对目标物的吸附机理不同,为了保证每种材料都能得到最好的脱附结果,使用了3种不同类型的脱附溶剂(甲酸-甲醇-水(0.5∶49.5∶50, v/v/v)、氨水-甲醇-水(0.5∶49.5∶50, v/v/v)、甲醇-水(50∶50, v/v))对萃取后的涂层纤维进行脱附。在添加有200 ng/mL的NE、EP、DA和5-HT, 20 μg/mL的GABA、Glu和Cho的脑替代基质中进行优化实验,静态萃取10 min后,用300 μL水冲洗10 s,随后将其浸泡于100 μL的脱附溶剂中超声10 min以解吸目标神经递质。结果如图3所示,当氨水-甲醇-水(0.5∶49.5∶50, v/v/v)为脱附溶剂时,COF-SO_3_H萃取神经递质的相对含量较HLB、C18、MCX、Amino要高,但低于PXC;当脱附溶剂为甲醇-水(50∶50, v/v)时,COF-SO_3_H纤维萃取神经递质的相对含量较HLB、C18、Amino要高,略低于PXC和MCX;当脱附溶剂为酸性的甲酸-甲醇-水(0.5∶49.5∶50, v/v/v)时,COF-SO_3_H纤维萃取效果最好。选用酸性脱附溶剂还可避免儿茶酚胺类神经递质(NE、EP、DA和5-HT)被氧化,增加目标化合物的稳定性,同时防止目标分析物穿透涂层表面的保护层并被反萃取。因此,COF-SO_3_H纤维在满足目标分析物稳定性的同时比其他萃取纤维萃取效果更好。

**图 3:**
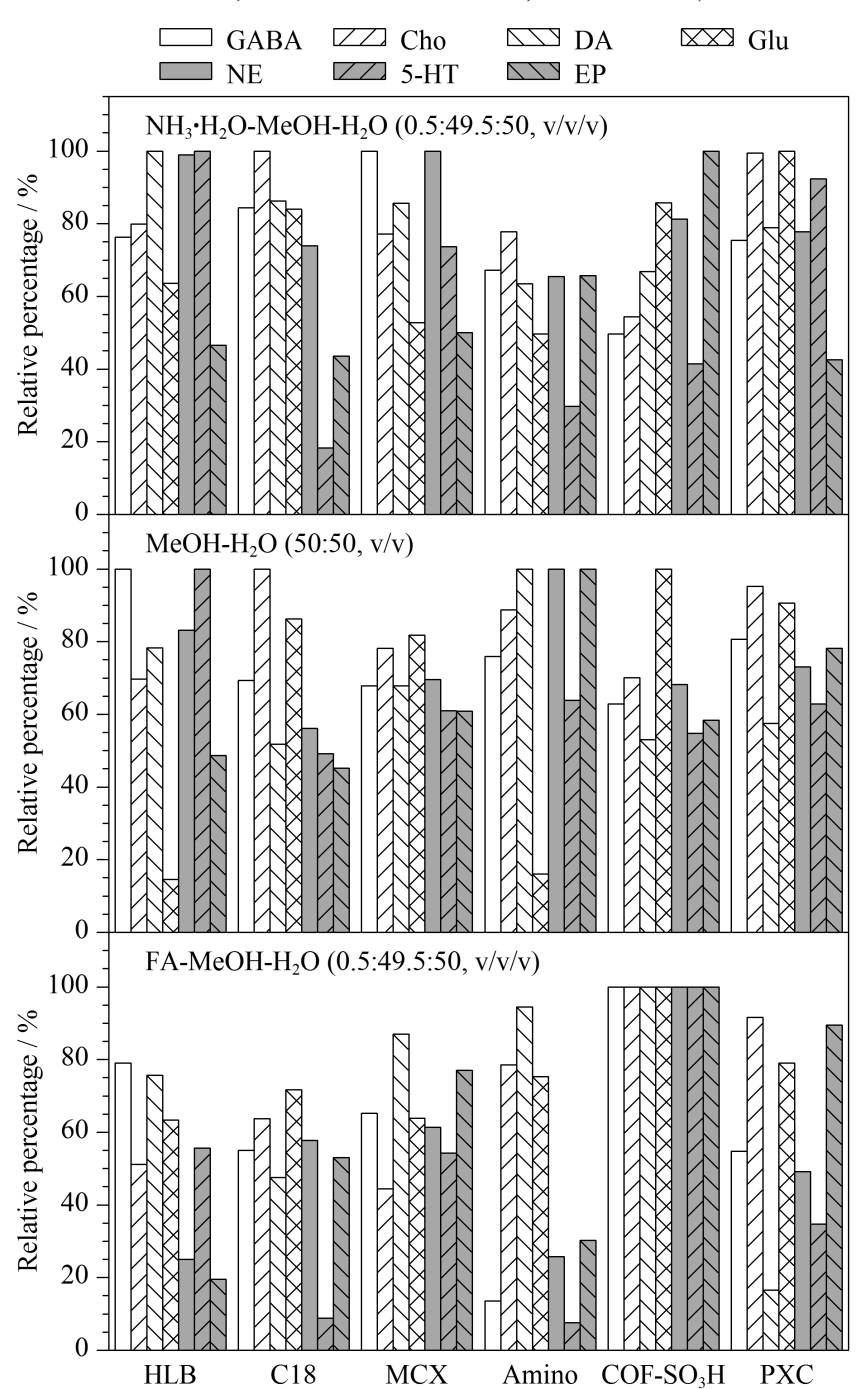
微萃取纤维对神经递质萃取量的影响

#### 2.2.2 脱附溶剂的优化

为了达到COF-SO_3_H纤维最好的萃取效果,优化了7种不同类型的酸性脱附溶剂,分别是甲酸-水-甲醇-乙腈(0.4∶39.6∶30∶30, v/v/v/v)、1%乙酸水溶液、1%甲酸水溶液、乙酸-水-甲醇-乙腈(0.4∶39.6∶30∶30, v/v/v/v)、1%甲酸甲醇溶液、1%乙酸甲醇溶液和甲酸-甲醇-水(0.5∶49.5∶50, v/v/v)。结果如图4a所示,脱附溶剂为甲酸-甲醇-水(0.5∶49.5∶50, v/v/v)时,脱附效果最好。因此,后续实验用甲酸-甲醇-水(0.5∶49.5∶50, v/v/v)来解吸COF-SO_3_H纤维上的神经递质。

**图 4:**
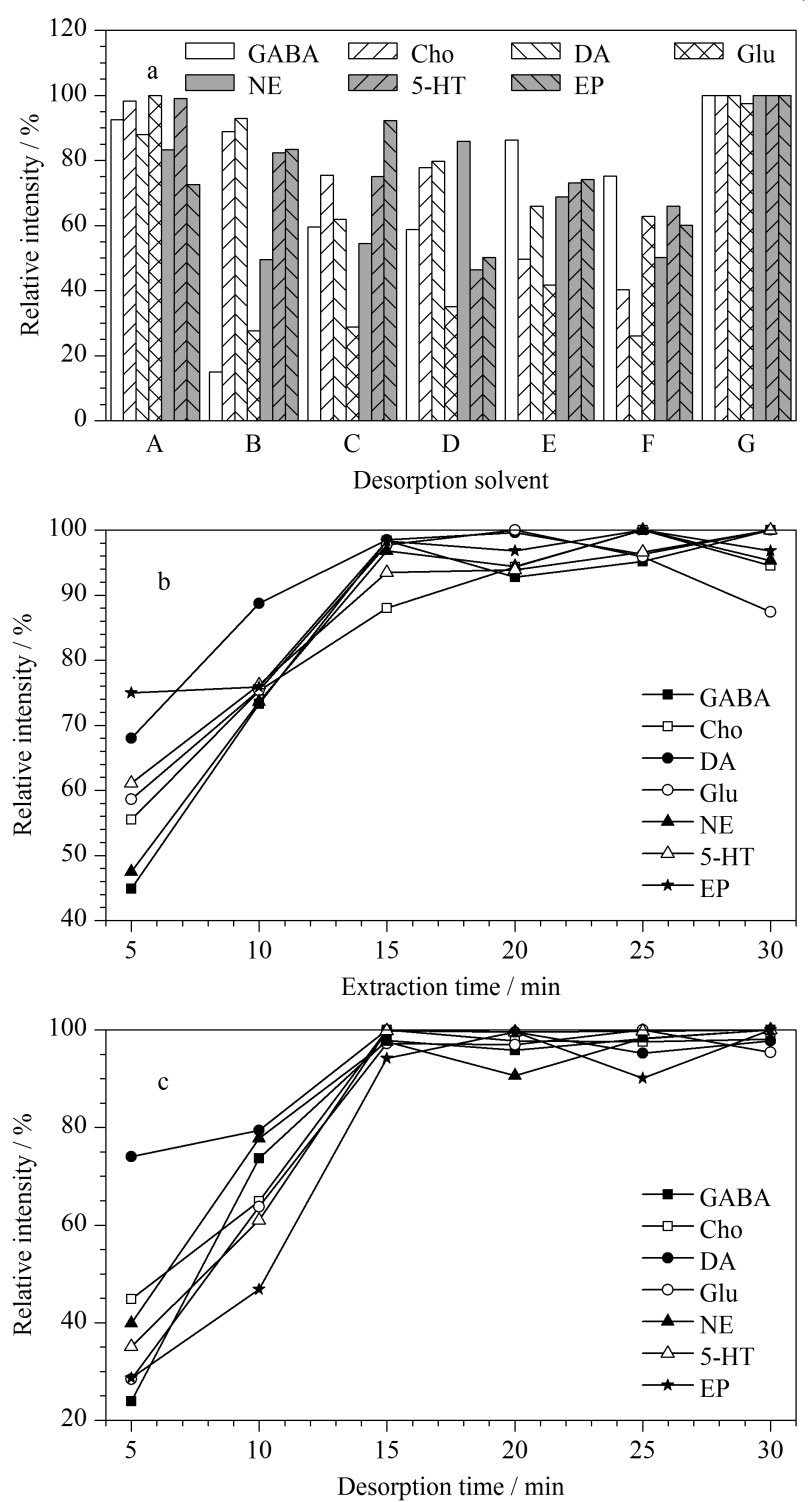
(a)脱附溶剂、(b)萃取时间和(c)解吸时间的优化

#### 2.2.3 萃取时间和解吸时间的优化

萃取时间和解吸时间是SPME中的关键因素,会显著影响萃取效率。为了确定和选择最佳平衡萃取时间,采用脑替代基质进行萃取时间和解吸时间的优化,如图4b所示,在5~15 min内,纤维上萃取神经递质的量随着萃取时间的增加而增加,15 min后萃取量处于动态平衡状态。在5~30 min内对解吸时间进行了优化,如图4c所示,随着解吸时间的增加,目标物的萃取量迅速增加并趋于平衡,在15 min后洗脱量基本保持不变。因此,以萃取时间15 min和解吸时间15 min进行后续实验。

### 2.3 方法验证

在最佳的萃取条件下,通过在500 μL的小鼠脑替代基质中加入质量浓度为1~3000 μg/mL的GABA、Glu和Cho,以及0.001~2 μg/mL的NE、EP、DA和5-HT来考察方法的线性。在所有样品中DA-d_4_和L-Glu-^15^N的质量浓度保持在20 ng/mL,Cho-d_9_和GABA-d_6_保持在1.25 μg/mL。通过目标分析物的质量浓度与其峰面积和内标物峰面积之比的关系,获得校准曲线。如表2所示,所有标准曲线具有良好的线性相关性(相关系数*r*^2^均大于0.99),单胺类神经递质和氨基酸类神经递质的定量限(*S/N*≥5)^[25]^分别为0.003~0.005 μg/mL和3~5 μg/mL,一日内连续制备4组同一质量浓度的样品进行分析,4次检测的RSD低于20%。以每个水平下的3次重复测量的RSD值评估该方法的精密度,以|(实测分析物浓度-理论加标浓度)/理论加标浓度|×100%计算准确度^[25]^,用下列公式计算绝对基质效应^[25]^:

AME= $\frac{C_{a+b}-C_{a}}{C_{b}}$ ×100%

其中,*C*_a+b_ (ng/mL)为加标小鼠脑匀浆样品中分析物的实测含量,*C*_a_ (ng/mL)为脑匀浆样品中分析物的含量,*C*_b_ (ng/mL)为纯解吸溶剂中加标分析物的实测含量。

**表 2:** 7种NTs的线性范围、线性方程、定量限和相关系数

Analyte	Linear range/ (μg/mL)	Linear equation	LOQ/ (μg/mL)	r^2^
GABA	1-3000	y=1.02×10^-2^x-3.84×10^-2^	5	0.9995
Cho	1-3000	y=1.00×10^-2^x-2.09×10^-2^	5	0.9996
Glu	1-3000	y=1.25×10^-2^x+2.99×10^-1^	3	0.9991
DA	0.001-2	y=8.70×10^-3^x+6.71×10^-1^	0.003	0.9988
NE	0.005-2	y=3.00×10^-3^x+2.31×10^-1^	0.005	0.9964
5-HT	0.001-2	y=2.40×10^-3^x+6.09×10^-2^	0.003	0.9987
EP	0.005-2	y=2.90×10^-3^x+2.18×10^-1^	0.005	0.9971

*y*: ratio of peak area of the target analyte to the peak area of the internal standard; *x*: mass concentration, μg/mL.

如表3所示,该方法的精密度(0.80%~9.50%)和准确度(2.08%~17.72%)良好,绝对基质效应为82.22%~117.92%,表明复杂基质对目标分析物的准确测定影响较小。

**表 3:** 7种NTs在小鼠脑匀浆中3个水平下的准确度、精密度和绝对基质效应(*n*=3)

Analyte	Accuracies/%				RSDs/%				AMEs/%		
	Low	Medium	High		Low	Medium	High		Low	Medium	High
GABA	13.83	6.68	3.40		0.80	1.68	7.66		85.04	117.92	89.22
Cho	17.58	8.17	2.52		1.84	2.58	7.25		101.63	108.13	102.22
Glu	9.38	7.31	2.08		5.21	3.96	5.99		90.28	97.29	91.46
DA	8.12	7.90	15.75		7.59	9.50	5.65		111.71	82.22	110.66
NE	14.79	6.21	17.72		1.81	2.88	5.87		105.46	96.35	108.99
5-HT	6.21	9.69	15.86		3.05	1.35	1.33		84.78	87.19	107.28
EP	10.55	8.16	8.20		1.67	1.69	3.25		89.20	100.60	104.91

AME: absolute matrix effect. The low, medium, and high levels for GABA, Cho, and Glu were 50, 200, and 1000 μg/mL, for DA, NE, 5-HT, and EP were of 50, 200, and 1000 ng/mL, respectively.

### 2.4 方法应用

为了评估COF-SO_3_H萃取纤维的实用性,将该纤维应用于测定小鼠脑部海马区的神经递质含量。使用1.5节的方法对小鼠进行造模和留置导管后,第2周开始对小鼠脑部海马区的神经递质进行分析,结果如图5所示。从第2周开始,与对照组相比,模型组小鼠脑内海马组织中神经递质含量明显降低;与模型组相比,随着给药时间周期的增加,天麻素治疗组可明显升高小鼠脑部海马区内神经递质的含量。结果表明,所建立的活体SPME-UPLC-MS/MS的分析方法为活体神经递质的富集分析提供了一种切实可行的方案。其次,天麻素对阿尔兹海默症具有潜在的治疗效果,为阿尔兹海默症的临床治疗提供了参考。

**图 5:**
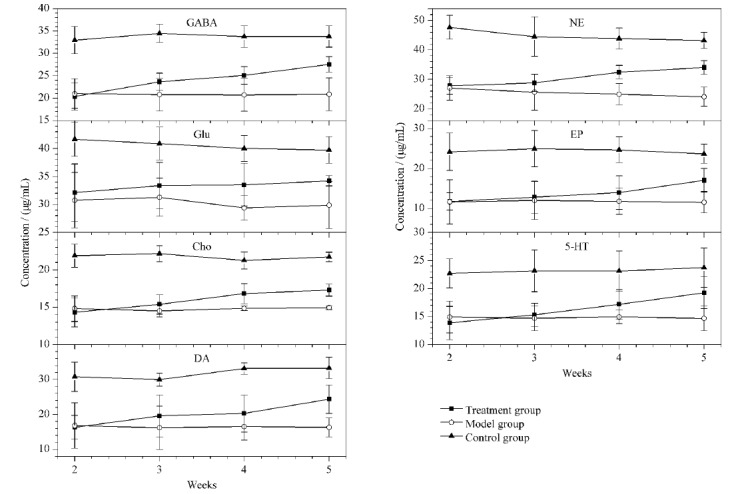
小鼠脑部海马区神经递质的含量变化(*n*=3)

## 3 结论

在室温下制备了具有强阳离子交换作用的COF-SO_3_H吸附材料,并将其制备成SPME纤维,结合UPLC-MS/MS用于小鼠脑部神经递质的检测。与其他5种商品化的纤维材料相比,COF-SO_3_H具有较好的吸附效果,这可归因于其独特的孔隙结构和丰富的基团,以及*π-π*作用和离子交换作用等。为了确保其在小鼠脑部应用的准确性,选择脑替代基质进行萃取条件的优化。在最优的萃取条件下,基于COF-SO_3_H的SPME-UPLC-MS/MS方法精密度良好,准确度高。此外,COF-SO_3_H具有较好的生物相容性,不会引起毒性反应和副作用,在留置导管的帮助下,可成功应用于小鼠脑部海马区神经递质的检测。

## References

[1] Qiao J, Wu D, Song Y Y, et al. Anal Chem, 2021, 93(44): 14743 34709796 10.1021/acs.analchem.1c03263

[2] Su Y, Bian S, Sawan M. Analyst, 2020, 145(19): 6193 32808603 10.1039/d0an01175d

[3] Godage N H, Olomukoro A A, Emmons R V, et al. TrAC-Trends Anal Chem, 2021, 142: 116290

[4] Gopinath A, Mackie P M, Phan L T, et al. Neurobiol Dis, 2023, 176: 105940 36470499 10.1016/j.nbd.2022.105940 PMC10372760

[5] Van Erum J, Van Dam D, De Deyn P P. Neurosci Biobehav Rev, 2019, 105: 72 31377219 10.1016/j.neubiorev.2019.07.019

[6] Arning E, Wasek B, Bottiglieri T. Methods Mol Biol, 2022, 2546: 165 36127587 10.1007/978-1-0716-2565-1_15

[7] Lakard S, Pavel I A, Lakard B. Biosensors, 2021, 11(6): 179 34204902 10.3390/bios11060179 PMC8229248

[8] Bai J, Wang D, Liu Z P, et al. Chinese Journal of Chromatography, 2020, 38(8): 923 34213184 10.3724/SP.J.1123.2019.12029

[9] Lendor S, Hassani S A, Boyaci E, et al. Anal Chem, 2019, 91(7): 4896 30848885 10.1021/acs.analchem.9b00995

[10] Matys J, Gieroba B, Józwiak K. J Pharm Biomed Anal, 2020, 180: 113079 31896524 10.1016/j.jpba.2019.113079

[11] Zhang W M, Li Q Q, Fang M, et al. Chinese Journal of Chromatography, 2022, 40(11): 1022 36351811 10.3724/SP.J.1123.2022.05001 PMC9654618

[12] Bojko B, Looby N, Olkowicz M, et al. J Pharm Anal, 2021, 11(1): 37 33717610 10.1016/j.jpha.2020.08.011 PMC7930785

[13] Napylov A, Reyes-Garces N, Gomez-Rios G, et al. Angew Chem Int Ed Engl, 2020, 59(6): 2392 31697450 10.1002/anie.201909430

[14] Reyes-Garcés N, Diwan M, Boyaci E, et al. Anal Chem, 2019, 91(15): 9875 31265251 10.1021/acs.analchem.9b01540

[15] Aslam M, Feleder C, Newsom R J, et al. Bioanalysis, 2019, 11(16): 1523 31486681 10.4155/bio-2019-0144 PMC6770421

[16] Sun Q, Ma W, Dan O, et al. Analyst, 2021, 146(9): 2991 33949450 10.1039/d1an00282a

[17] Jiang S Y, Gan S X, Zhang X, et al. J Am Chem Soc, 2019, 141(38): 14981 31492052 10.1021/jacs.9b08017

[18] Zhao Y Q, Hu K, Yang C, et al. Anal Chim Acta, 2023, 1237: 340581 36442935 10.1016/j.aca.2022.340581

[19] Shi Y, Hu K, Cui Y X, et al. Microchem J, 2019, 146: 525

[20] Li H, Ren G B, Li H J, et al. Chinese Journal of Chromatography, 2022, 40(8): 704 35903837 10.3724/SP.J.1123.2022.03017 PMC9404133

[21] Zhuang S, Liu Y, Wang J. J Hazard Mater, 2020, 383: 121126 31505428 10.1016/j.jhazmat.2019.121126

[22] Wang Y, Wu S, Wu D. Anal Chim Acta, 2020, 1093: 61 31735216 10.1016/j.aca.2019.09.078

[23] Zhou Y, Liu S, Hu C, et al. J Mater Chem B, 2020, 8(25): 5451 32459249 10.1039/d0tb00679c

[24] Ma W, Zheng Q, He Y, et al. J Am Chem Soc, 2019, 141(45): 18271 31656073 10.1021/jacs.9b09189

[25] Lendor S, Hassani S A, Boyaci E, et al. Anal Chem, 2019, 91(7): 4896 30848885 10.1021/acs.analchem.9b00995

